# What Do Introduction to Psychology Textbooks Have to Say About Older Adults?

**DOI:** 10.1093/geroni/igab046.2796

**Published:** 2021-12-17

**Authors:** Zoe Hancock, Matthew Wynn, Brian Carpenter

**Affiliations:** 1 Washington University in St. Louis, Saint Louis, Missouri, United States; 2 Washington University in St. Louis, Washington University in St. Louis, Missouri, United States

## Abstract

Introduction to Psychology is one of the most popular undergraduate courses, an entry course for psychology majors and also popular with students from other disciplines. Consequently, the content in introductory psychology textbooks has the potential to influence undergraduates’ knowledge, attitudes, and interests, including those related to aging. The purpose of this study was to analyze aging-related content in introductory psychology textbooks to understand the topics to which students are exposed in this important course. We analyzed the indices of 21 best-selling Introduction to Psychology textbooks for both advanced and intermediate audiences, published between 2018 and 2020. We extracted and aggregated 275 unique, aging-specific index terms from the textbooks and analyzed their relative frequency. We identified 61 superordinate index terms corresponding to general terms (e.g., “aging,” “death”). The indices also included 214 unique subordinate terms that were more specific (e.g., “aging, and cognition”). Across textbooks, the most frequent topics reflected negative consequences of aging (e.g., “Alzheimer disease” = appeared in 100% of textbooks, “death” in 52%). In contrast, positive aspects of aging appeared less often (e.g., “generativity” in 47%, “longevity” in 10%). Terms describing career opportunities were rare (e.g., “gerontology” in 5%, “geropsychology” in 5%), as were modern theories (e.g., “socioemotional selectivity theory” in 28%). Advocacy for comprehensive and balanced representation of aging in introductory psychology textbooks is critical for educating students and promoting interest in the field.

